# Comparison of the Effect of 8 weeks Aerobic and Yoga Training on Ambulatory Function, Fatigue and Mood Status in MS Patients

**DOI:** 10.5812/ircmj.3597

**Published:** 2013-06-05

**Authors:** Azra Ahmadi, Ali Asghar Arastoo, Masoud Nikbakht, Shahla Zahednejad, Mojtaba Rajabpour

**Affiliations:** 1 School of Physical Education and Sports Science, Ahvaz Shahid Chamran University, Ahvaz, IR Iran; 2 Physiotherapy Group, School of Rehabilitation Sciences, Ahvaz Jundishapur University of Medical Sciences, Ahvaz, IR Iran; 3 Sport Physiology Group, School of Physical Education and Sports Science, Ahvaz Shahid Chamran University, Ahvaz, IR Iran; 4 Physiotherapy Group, School of Rehabilitation Sciences, Musculoskeletal Rehabilitation Research Center, Ahvaz Jundishapur University of Medical Sciences, Ahvaz, IR Iran; 5 School of Psychology, Semnan University, Semnan, IR Iran

**Keywords:** Multiple Sclerosis, Rehabilitation, Exercise, Fatigue

## Abstract

**Background:**

Multiple Sclerosis (MS) is a disease of the central nervous system that results in many symptoms including mobility limitation and fatigue.

**Patients and Methods:**

Thirty-one MS patients, all female with mean of age of 36.75 years and Expanded Disability Status Scale scores (EDSS) of 1.0 to 4.0 were recruited. Subjects were randomly assigned to one of the three groups: treadmill training, yoga or control groups. Treadmill training and yoga practice consisted of 8 weeks (24 sessions, thrice weekly). The control group followed their own routine treatment program. Balance, speed and endurance of walking, fatigue, depression and anxiety were measured by Berg Balance scores, time for 10m walk and distance for a two minute walk, Fatigue Severity Scale (FFS), Beck Depression Inventory (BDI) and Beck Anxiety Inventory (BAI), respectively.

**Results:**

Comparison of results have shown that pre- and post-interventions produced significant improvements in the balance score, walking endurance, FFS score, BDI score and BAI score in the treadmill training group and yoga group. However, 10m walk time decreased in the treadmill training group but did not show any clear change in the yoga group. Moreover, the analysis showed significant differences between the treadmill training group and yoga group for BAI score.

**Conclusions:**

These results suggest that treadmill training and yoga practice improved ambulatory function, fatigue and mood status in the individuals with mild to moderate MS.

## 1. Background

Today, exercise is recommended for the treatment of a large number of diseases such as metabolic syndrome related disorders, heart and pulmonary diseases, muscle, bone and joint diseases (1). Multiple sclerosis (MS) is an autoimmune disease and a progressive demyelinating disease that affects the white matter of the central nervous system (2). MS patients suffer from a range of symptoms including: loss of function, fatigue, muscular weakness, spasticity, ataxia, cognitive dysfunction and depression (2, 3). It is estimated that between 65 - 87% of people with MS have some form of balance or mobility impairment impacting the quality of life of people with MS (4). Among various symptoms, reduced mobility and fatigue are the most important problems. Nearly 85% of individuals with MS reported difficulty in walking (5). In addition, recent evidence suggested that gait altered by this disease can lead to increase of fatigue (5). In MS individuals, depression, anxiety and stress has been shown to be greater than in healthy individuals (6). Some studies found a relationship between involvement of specific areas of the brain and the occurrence of depression and anxiety (7). Studies showed that nearly 50 - 60% of MS patients reported depression (8) and about 25 - 40% experienced anxiety (9), affecting quality of life in these patients. One of the solutions for MS patients is the application of pharmacological treatment, which may not be effective for all patients with MS. Moreover, the associated prescribed drugs have many side effects such as fatigue and psychological imbalance (10). For instance, regulator drugs of immune system and steroid therapy can reduce some symptoms of MS and are effective and widely used in patients, but these drugs cannot stop the progressive disease course and also have numerous side effects such as increased spasm, nausea, depression, nerve pain, fever, headache, etc. (11). Therefore, non-pharmacological methods known as complementary therapies have emerged in recent years for MS patients. Complementary therapies are treatments with a comprehensive nature that is used for physical and mental comfort of patients (12). Complementary therapies have many benefits for patients with multiple sclerosis and have been widely considered for these patients (10). Exercise is a complementary treatment that is suggested for these patients (13, 14). However in the past, physicians had limited knowledge about physical activity and believed that fatigue and overheating problems associated with the disease would exacerbate the symptoms of MS (8). But recently, some researchers have demonstrated that exercise is a low cost form of therapy that has achieved recognition as a realistic and positive method for MS patients. As an example, Mostert and Kesselring (2005) reported improvement in aerobic threshold, work rate and activity level of patients after four weeks of aerobic exercise training (2). In addition, treadmill training, aquatic, cycling and resistance training may be beneficial for patients with MS (15-19). Moreover, cognitive changes (e.g. quality of life, fatigue, and mood) are often present in MS patients that may be alleviated by yoga or physical activity (20). Yoga is an ancient Indian science and its practice is known to improve physical and mental health status by improving cardio-vascular, cardio-respiratory and other functional capabilities and also prevents various sufferings (21).Yoga postures may be used with great success to decrease fatigue and spasticity, to promote muscle relaxation and to improve mood (20).

## 2. Objectives

The purpose of this study was the comparison of the effect of eighth weeks of aerobic and yoga training on balance, ambulatory function, fatigue and mood status of MS patients.

## 3. Patients and Methods

Patients: 31 women with MS (19 - 54years old) were screened from a waiting list for a rehabilitation program in the Physiotherapy Clinic of the Jundishapour University of Medical Sciences, Iran, whom obtained physician clearance prior to study enrolment. Subject inclusion criteria consisted of physician diagnosed MS with a self-assessed Kurtzke’s Expanded Disability Status Scale (EDSS) score of between one and four. In addition, individuals were required to be able to walk on the treadmill with or without hand support (without human assistance) and to be able to walk at a constant speed on a treadmill for five minutes. No subjects had participated in any physical activity for at least three months prior to the study. Subjects using MS disease modifying drugs were also included. It should be mentioned that individuals who had cardiovascular disease, liver or kidney failure, symptomatic lung disease, diabetes, thyroid disorders, gout or orthopedic limitations were excluded. Pregnant and addicted individuals (i.e. cigarette smokers or drug addicts) were also excluded. All of the subjects provided a written informed consent for the study. After completion of the baseline evaluations, subjects were randomized to one of the three experimental groups lasting eight weeks: (i) treadmill training, (ii) yoga practice, or (iii) wait-list control groups. Subject characteristics are presented in Table 1. 

**Table 1.** Characteristics of 31 Subjects With Multiple Sclerosis Enrolled in the Study.

Variable	All Subjects, n = 31	Treadmill training group, n = 10	Yoga group, n = 11	Control group, n = 10	P^a^
**Age, y^b^, range**	35.16 ± 9.01, 19 - 54	36.80 ± 9.17, 24 - 54	32.27 ± 8.68, 22 – 53	36.70 ± 9.32, 19 - 49	0.43
**Disease duration, y, range**	5.09 ± 4.09, 1 - 20	5.60 ± 3.30, 2 - 11	4.72 ± 5.62, 1 - 20	5.00 ± 3.05, 1 - 9	0.89
**EDSS score (0 – 10), range**	2.20 ± 1.16, 1 - 4	2.40 ± 1.24, 1 - 4	2 ± 1.09, 1 - 4	2.25 ± 1.25, 1 - 4	0.74
**Balance score, range**	46.29 ± 7.50, 28 - 56	46.20 ± 6.32, 35 - 56	47.72 ± 6.78, 34 - 54	44.50 ± 9.43, 28 - 54	0.62
**Walk time, s** ^**b**^ **, range**	8.85 ± 1.82, 6.19 - 12.24	8.68 ± 1.93, 6.19 - 12.01	8.78 ± 1.79, 7.20 - 11.85	9.16 ± 1.88, 6.62 - 12.01	0.80
**Walk distance, m ** ^**b**^ **, range**	117.37 ± 22.5, 71 - 172	120.40 ± 20.29, 89 - 146	109.45 ± 17.44, 85 - 13	121.50 ± 27.73, 71 -17	0.36
**FFS score, range**	3.85 ± 1.35, 1 - 5.78	3.46 ± 1.77, 1 - 5.33	3.98 ± 0.99, 2.11 - 5.33	4.17 ± 1.28, 1.11 - 5.78	0.40
**BDI score, range**	12.74 ± 9.73, 3 - 45	8.50 ± 3.06, 3 - 13	3.98 ± 0.99, 2.11 - 5.33	11.90 ± 9.39, 3 - 35	0.10
**BAI score, range**	9.38 ± 6.04, 1 - 22	6.10 ± 4.95, 0 - 17	12.45 ± 4.54, 7 - 20	7.50 ± 6.77, 1- 22	0.10

^a^ P: P values assessed by multivariate test (MANOVA)

^b^ Abbreviations: y, year; s, second; m, meter

After medical history screening, participants were asked to complete the FFS15Beck Depression Inventory (BDI) (20), and Beck Anxiety Inventory (BAI) (22), in order to assess their level of fatigue, depression, and anxiety. Balance and some walking parameters were evaluated as indicators of ambulatory function (23). Balance was assessed using the Berg Balance Scale (23), and the 10 m walk and two minute walk, evaluated walking speed and endurance, respectively (15). The time taken to walk 10 m over a straight path was recorded, as well as, the distance travelled walking for two minutes around a shuttle corridor track. Individuals were familiarized with the treadmill and all test protocols. All participants were then assessed immediately prior to baseline, followed by the eight weeks intervention.

Treadmill training subjects completed supervised treadmill training (thrice weekly) exercises for eight consecutive weeks. Each training session consisted of 30 minutes of treadmill exercise. The exercise class began and ended with about 10 minutes of stretching of muscles and flexion and rotation movements of the trunk and the lower limb. Training intensity was 40-75% age predicted maximal heart rate, which was measured on a Polar Electro OY type PE-3000 heart rate monitor. Initial speed was based on baseline comfortable walking speed and was increased as directed by participants. To monitor exercise intensity, HR, time, speed and ratings of perceived exertion were recorded using the modified Borg 15 -point scale. Eleven patients in the yoga practice group completed an eight week yoga class. Yoga classes were 60 - 70 minutes in duration and three sessions per week. Hatha yoga has three basic components, postures (asanas), breathing techniques (pranayama) and meditation (dhyana). The postures started with stretching techniques followed by standing, supine and prone-lying and sitting postures. Our yoga teacher was familiar with problems common to people with MS. In this regard, she employed previous studies to design a Hatha yoga program with the following techniques: breathing techniques, arms overhead stretches (static), eagle pose (garudâsana), side bending triangle posture (trikonasana), forward bending (padahastasana), side lateral bending (ardhakati chakrasana), ankle on knee forward bend, warrior II (vîrabhadrâsana II) on chair, side angle pose (parshvakonâsana), seated twist (bharadvajâsana I), tree pose supported by wall (vrikshâsana), reclining bound angle (suptabaddha-konâsana), supported downward dog (adhomukha shvanâsana), cat pose, pose of a child (balâsana), hand to toe (supta-padângushthâsana), supported back bend, rising sun twist, variation of jathara-parivartanâsana, legs up the wall (viparîta-karanîmudrâ) and followed by a relaxation technique in the supine posture with closed eyes and relaxation of every part of the body (20, 24-26). Each pose was held for approximately 10 - 30 seconds (even eight seconds for subjects who were unable to maintain some techniques) with resting periods between poses lasting 30 seconds to one minute. Patients were supported for the majority of poses, with a chair, Swiss ball or wall. Usually, classes began with a calmative music. The yoga class was set up in a physiotherapy clinic and supervised by a neurologist and a physiotherapist. Since overheating problems associated with the disease would aggravate the symptoms of MS, temperature was kept about 23 - 26 °C in the training time. Statistical analyses: comparisons between pre- and post-training were analyzed using a paired t-test for within-group differences. In this study, analysis of variables was performed using the MANOVA and Tukey test procedures for between-group differences. Statistical analysis was carried out using the SPSS version 16.0 software, with a significance level of p < 0.05.

## 4. Results

A total number of ten MS women in the treadmill training group, eleven patients in the yoga group and ten MS women in the control group took part in this study. No subject exacerbations were reported during the eight week training program. There were no differences between the groups at baseline for age, EDSS score, disease duration, balance score, walk time, walk distance, FFS score, BDI score and BAI score, as shown in Table 1. Balance score was increased significantly by 16.45% (p = 0.001) and 12.76% (p = 0.001) in the treadmill training and yoga groups, respectively, and decreased insignificantly by 6.29% (p = 0.07) in the control group after 8 weeks. The analysis did not show any difference between treadmill training group and yoga group regarding balance score (p = 0.76). In the treadmill training group, the mean 10m walk time decreased by 18.54% (P = 0.001), whereas changes of this factor in the yoga practice and control groups were not significant. In the yoga practice group, the mean 10m walk time decreased by 7.40% (p = 0.13), whereas this increased in the control group by 3.38% (P = 0.14). When interventions were analyzed between groups, mean 10m walk time after eight weeks in the treadmill training group was significantly increased compared with the control group (p = 0.001). However, there was no significant difference between treadmill training group and yoga practice group (p = 0.12). Mean two minutes’ walk distance increased significantly in the treadmill training group and yoga practice group by 16.19% (p = 0.001) and 9.96% (P = 0.001), respectively; and remained almost unchanged in the control group with a 2.01% decline (P = 0.15). However, no difference was observed between treadmill training group and yoga group for mean two minutes’ walk distance (p = 0.26). For evaluation of the level of excessive fatigue, the FFS score was used in this study. It was observed that the fatigue levels were significantly lower during post-test than pre-test in the treadmill training and yoga groups by 45.08% (p = 0.001) and 38.69% (p = 0.01), respectively, with a negligible increase of 1.43% (p = 0.82) in the control group. However, no significant difference was observed between the treadmill training group and yoga practice group (p = 0.99). After eight weeks, BDI score improved significantly in the treadmill training group (p = 0.001) and decreased by 34.11%; whereas, BAI score reduced significantly by Beck et al. (1996) 78% (p = 0.01). In the yoga practice group, average BDI score and BAI score decreased significantly by 36.11% (p = 0.001) and 48.56% (p = 0.001), respectively. Both BDI score and BAI score increased in the control group. When interventions were analyzed between groups, BAI score after 8 weeks improved in the yoga practice group more than the treadmill training group, and this difference was significant (p = 0.01). The analysis did not show any differences between treadmill training group and yoga practice group for BDI score (p = 0.11), as shown in Tables 2 and 3. 

**Table 2.** Pre-intervention and Post-intervention Values for Balance Score, 10m Walk Time (Second), Two Minute Walk Tests (Meter), Fatigue Scale Score, BDI Score and BAI Score in Subjects With Multiple Sclerosis

Variable	Treadmill training group			Yoga group			Control group			F	P^b^
	Pre	post	P^a^	Pre	post	P^a^	Pre	post	P^a^		
**Balance score, range**	46.20 ± 6.32, 35-56	53.80 ± 2.34, 50-56	0.001	47.72 ± 6.78, 34-54	53.81 ± 3.40, 45-56	0.001	44.50 ± 9.43, 28-54	41.70 ± 8.48, 28-54	0.07	14.62	0.001
**Walk time, range**	8.68 ± 1.93, 6.19 - 12.01	7.07 ± 1.03, 5.84 - 8.45	0.001	8.78 ± 1.79, 7.20 - 11.85	8.13 ± 1.87, 6.12 - 11.25	0.13	9.16 ± 1.88, 6.62 - 12.01	9.47 ± 1.92, 7 - 12.75	0.14	8.02	0.001
**Walk distance, range**	120.40 ± 20.29, 89-146	139.90 ± 20.78, 116.50 - 184	0.001	109.45 ± 17.44, 85 - 133	120.36 ± 20.62, 86 - 162.50	0.001	121.5±27.73, 71-172	119.05±27.12, 73-154	0.15	10.01	0.001
**FFS score, range**	3.46 ± 1.77, 1 - 5.33	1.90 ± 0.73, 1 - 2.88	0.001	3.98 ± 0.99, 2.11-5.33	2.44 ± 1.50, 1.44 - 6.66	0.01	4.17 ± 1.28, 1.11 - 5.78	4.23 ± 1.04, 1.78 - 5.33	0.82	4.65	0.01
**BDI score, range**	8.50 ± 3.06, 3 - 13	5.60 ± 3.40, 1-10	0.001	17.36 ± 12.42, 4-45	11.09 ± 12.46, 1-39	0.001	11.90 ± 9.39, 3 - 35	12.50 ± 8.12, 4 - 30	0.53	8.80	0.001
**BAI score, range**	7.90 ± 5.91, 1 - 20	6.10 ± 4.95, 0 - 17	0.01	12.45 ± 4.54, 7 - 20	6.45 ± 3.61, 1 - 13	0.001	7.50 ± 6.77, 1 - 22	8.20 ± 7.39, 0 - 26	0.52	11.30	0.001

^b^ P: P values assessed by multivariate test (MANOVA)

^a^ P: P values assessed by paired t–test

**Table 3.** Mean Difference to Analyze Between Groups (Treadmill Training, Yoga and Control) by Tukey Test

Variable	Balance score		Walk time		Walk distance		FFS score		BDI score		BAI score	
	Mean Difference	P^a^	Mean Difference	P	Mean Difference	P	Mean Difference	P	Mean Difference	P	Mean Difference	P
**Treadmill training-Yoga**	1.40	0.76	0.95	0.12	8.89	0.26	0.05	0.99	3.37	0.11	4.20	0.01
**Treadmill training-Control**	10.30	0.001	1.91	0.001	25.15	0.001	1.64	0.03	3.50	0.11	2.50	0.22
**Yoga-Control **	8.89	0.001	0.96	0.11	16.25	0.01	1.59	0.03	6.87	0.001	6.70	0.001

^a^ P: P values assessed by paired Tukey test

## 5. Discussion

The results of this study revealed the fact that both treadmill training and yoga practice lead to significant and clinically meaningful changes in the balance of patients with mild to moderate MS. It is thought that the changes in balance and mobility accompanying yoga practice intervention occur due to the improvement of slow controlled movement in shifting the center of mass ( 4). Romberg et al. (2007) did not report any change in balance after a six months exercise program (strength training and aquatic training) for MS patients with mild to moderate disability (EDSS 1.0 to 5.5) (17). On the other hand, Giesser et al. (2007) showed improvements in balance after 40 treadmill training sessions by MS individuals with severely impaired ambulation (EDSS 7.0 to 7.5) (27). Following the eight week intervention, the treadmill training group showed significant improvements in the 10m walk time and the two minute walk. The findings of this study may be justified according to the principle of specificity (28) of the training program. In the Newman et al. study, the mean 10m walk time decreased by 12% relative to baseline after four weeks of treadmill training (15). Training appears to have benefited individuals by reducing the energy expenditure during walking. Even small savings in energy for those with more restricted mobility could be functionally important, allowing them to be active for longer periods of time (15). Our results showed that yoga practice does not lead to a significant improvement in 10m walk time after eight weeks. Perhaps, long-term yoga practice can be effective for improvement of this factor in MS patients. On the other hand, yoga practice intervention induced a significant change in the mean two minute walk. It has been reported that the practice of yoga improves VO2 (29), which would probably be beneficial for improvement of endurance in MS patients. Our findings from the FFS scale showed that treadmill training and yoga practice lead to a significant decrease of fatigue in MS patients. Likewise, some studies reported that various types of exercises can be useful to reduce fatigue in MS individuals (19 , 20 , 30). In contrast, the reported results of other exercise therapy programs did not show similar outcomes (15 , 17 , 19). The results of this study revealed that subjects had minimal to mild levels of depression and anxiety at the baseline (20 , 21). Besides, the level of disability of the participating subjects in this study was mild to moderate. Some studies indicated a correlation between anxiety and depression with level of disability in people with MS (7). Nevertheless depression is complex in nature and can be affected by different factors such as pain, sexual dysfunction, physical impairment, stress and drugs in MS patients (9). Our study revealed that eight weeks treadmill training improved BDI score in the same extent as yoga practice as shown in Table 2. Although the improvement in BDI score, resulting from treadmill training program and yoga practice was significant, the control group only showed minimal changes. Moreover, Aldridge et al. (2005) have shown that music therapy can lead to a decrease in depression and stress in individuals with MS (31), and this concept was considered for the yoga class of this study, which used a calmative music. Our findings in this study by the BAI scale showed that treadmill training and yoga practice lead to a significant decrease of anxiety in MS patients. This improvement in the yoga practice group was more than the treadmill training group (Table 2). According to Chaya et al. (2006), Hatha yoga improves mood and calms the mind and these can be considered as key indicators for assessment of improvements in the yoga practice group. Petajan et al. (1996) found reduced scores for anxiety and fatigue measured with the Profile of mood status in their training group after five weeks of bicycle exercise (8). Moreover, it is well known that in gregarious exercises such as yoga, other participates can be effective on changing of mood. Results of the present study clearly indicate that exercise therapy accompanied by drugs (treadmill and yoga groups) can be beneficial for individuals with MS. Both treadmill training group and yoga practice group showed improvements in balance, ambulatory function, fatigue and mood status; whereas the control group that followed their own routine treatment program did not show these developments (Table 3). Yoga practice can be attractive and calming for these patients and is also advantageous as a traditional exercise program e.g. treadmill training. There are some limitations associated with the current study. For instance, only women participated in this study, whereas it would be preferred if men also participated in separate groups. However, there were a few men available whom were excluded early in the study. We suggest that the same study should be conducted for men with MS. Lastly, it is suggested that the effects of other exercises should be investigated for treating MS symptoms. Results of the present study clearly indicated that exercise therapy accompanied by drugs could be beneficial for individuals with MS. In addition, treadmill training and yoga practice could improve ambulatory function, fatigue and mood status in individuals with mild and moderate MS. 
